# Supplementary material for: The interactome of intact mitochondria by cross-linking mass spectrometry provides evidence for coexisting respiratory supercomplexes
Source: Mol Cell Proteomics. 2017 Dec 8;17(2):216–32. doi: 10.1074/mcp.RA117.000470 (PMC5795388; doi:10.1074/mcp.RA117.000470)
Supplement: Supplemental Data [file supp_RA117.000470_133922_0_supp_23978_fzffwf.zip › spectra_annotation/mito_DR_spectra_annotation/395-1-13-1-5-1.pdf]

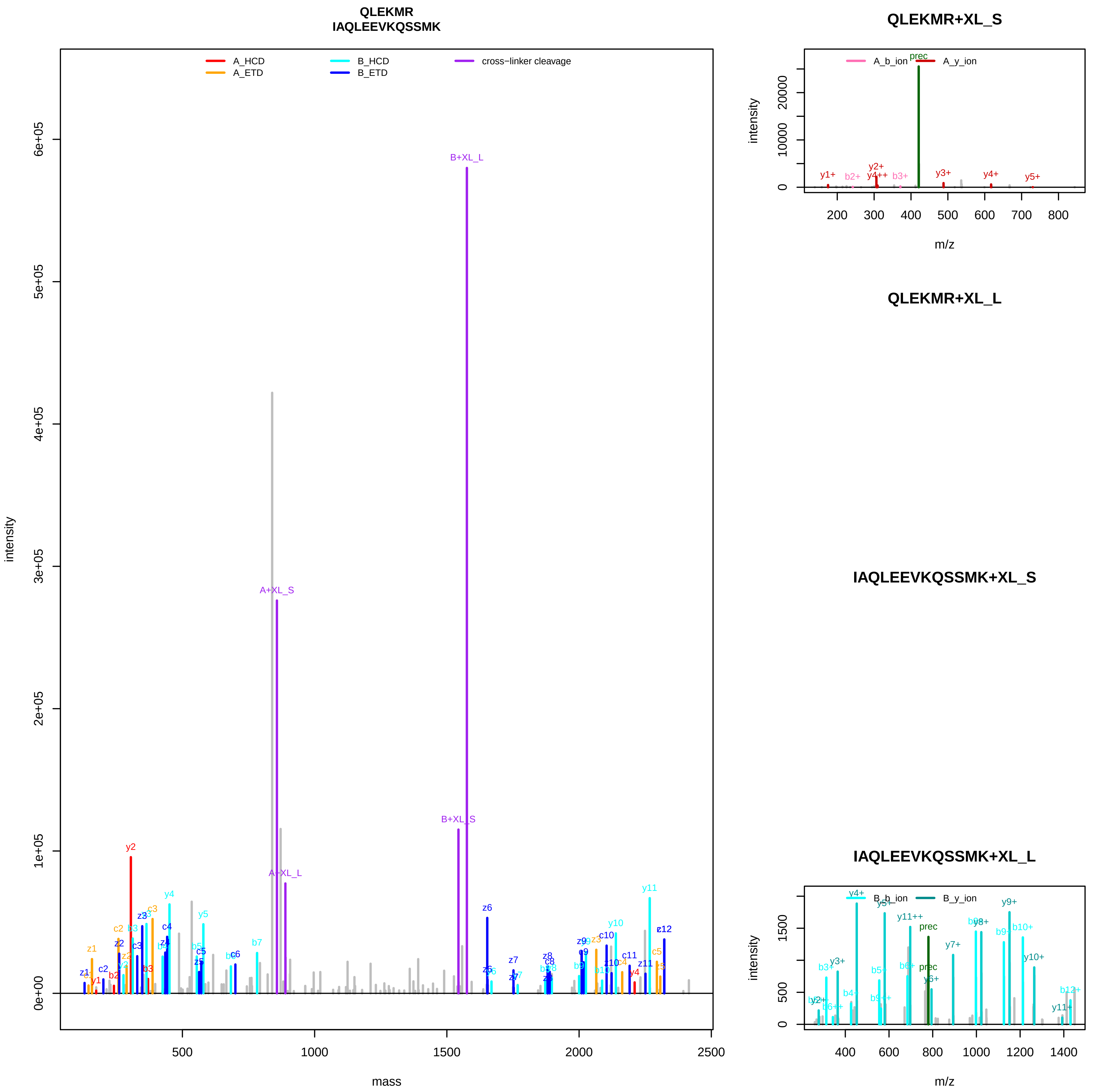
QLEKMR
IAQLEEVKQSSMK
A_HCD
A_ETD
B_HCD
B_ETD
cross−linker cleavage
B+XL_L
A+XL_S
B+XL_S
A+XL_L
y2
y4
y5
y10
y11
z6
c12
intensity
mass
0e+00
1e+05
2e+05
3e+05
4e+05
5e+05
6e+05
500
1000
1500
2000
2500
QLEKMR+XL_S
A_b_ion
A_y_ion
prec
y1+
b2+
y2+
y4++
b3+
y3+
y4+
y5+
intensity
m/z
0
10000
20000
200
300
400
500
600
700
800
QLEKMR+XL_L
IAQLEEVKQSSMK+XL_S
IAQLEEVKQSSMK+XL_L
B_b_ion
B_y_ion
y4+
y5+
y11++
prec
y9+
y8+
b10+
y7+
y10+
b12+
y11+
intensity
m/z
0
500
1500
400
600
800
1000
1200
1400
